# Integrating Single‐Cell and Spatial Transcriptomics Reveals NK Cell Subpopulations Associated With Immunotherapy for Melanoma

**DOI:** 10.1002/smmd.70023

**Published:** 2025-12-03

**Authors:** Zhicheng Hu, Yongfei Chen, Hao Yang, Qiuming Pan, Hongrui Li, Shuting Li, Junxi Wang, Yudi Huang, Guanglong Huang, Shanqiang Qu

**Affiliations:** ^1^ Department of Burn Surgery The First Affiliated Hospital of Sun Yat‐sen University Guangzhou China; ^2^ Department of Neurosurgery Nanfang Hospital Southern Medical University Guangzhou China; ^3^ The Laboratory for Precision Neurosurgery Nanfang Hospital Southern Medical University Guangzhou China

**Keywords:** biomarker, immunotherapy, NK cells, single‐cell sequencing, spatial transcriptome sequencing

## Abstract

Immune checkpoint inhibitors (ICI) have demonstrated prolonged efficacy in certain melanoma patients, yet a significant portion of patients do not experience clinical improvement, with the mechanisms underlying this resistance still not fully understood. Using established cell markers, we partitioned the single‐cell transcriptome into clusters, finding a notable link between NK cells and patient response to immunotherapy. We further identified four distinct subpopulations of NK cells, profiling marker gene sets and unique biological functions associated with each subpopulation. This analysis provides insights into the trajectory of NK cell development and differentiation, along with identifying the transcription factors driving these processes. The study pinpointed NK cluster 01 as pivotal in influencing patient sensitivity and prognosis during immunotherapy. Single‐cell transcriptome and spatial transcriptomics (ST) analysis revealed the proximity of NK cluster 01 cells to melanoma cells, hinting at a potential regulation of cell‐cell interaction via the IFN‐II signaling pathway network. ST analysis revealed the spatial arrangement and interaction of NK cluster 01 cells with melanoma cells. This study explores the feasibility of targeting NK cluster 01 cells with small molecule drugs via molecular docking, offering a promising approach to bolster the clinical utility of NK cell therapy. We comprehensively analyze the heterogeneity of NK cells within melanoma, elucidate the potential regulatory interactions between NK cells and other microenvironmental components, and establish a basis for the future clinical utilization of distinct NK cell subsets as therapeutic targets.

## Key Points

- Four NK cell subtypes have been identified and their biological roles were elucidated.
- NK cluster 01 and melanoma cells exhibit spatial interactions characterized by mutual repulsion.
- Specific NK cell subtypes as potential targets for immunotherapy in melanoma.
- A panel of five genes could be utilized to screen patients who would benefit from immunotherapy.

## Graphical abstract

We identified four distinct NK cell subtypes within the melanoma microenvironment and elucidated their biological roles. Crucially, spatial analysis demonstrated that a specific subtype (NK cluster 01) exhibits a mutual repulsion with melanoma cells, suggesting an active evasion mechanism. These insights highlight specific NK cell subtypes as promising potential targets.

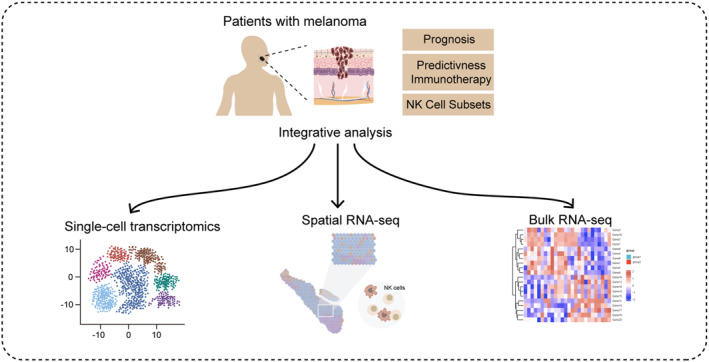

## 1 Introduction

Cutaneous melanoma (CM) is a deadly and aggressive malignancy with increasing incidence and mortality rates worldwide [1]. According to the World Cancer Research Fund International, 132,000 melanoma skin cancers are estimated to occur globally each year [2]. The incidence of CM in the United States is 31.91 per 100,000 individuals, with the annual incidence increasing at a rate of approximately 3.1% [3]. Therefore, the diagnosis and treatment of CM need to be enhanced to a greater extent. CM is characterized by an insidious onset, high degree of malignancy, rapid progression, early metastasis, and recurrence [4]. Current treatment options include surgical resection, chemotherapy, and targeted therapy [5]. However, even with standard management, the overall prognosis remains poor due to the tumor's high metastatic potential [6]. Adoptive immunotherapy for cancer has made significant progress in recent years, with impressive results achieved in patients with melanoma. However, the response rate to immune checkpoint‐blocking therapy differs among patients with melanoma, with a large proportion of those with advanced cancer failing to benefit from immunotherapy or developing tumor recurrence or further progression early in the treatment phase [7, 8]. Therefore, it is necessary to understand the mechanism of cellular and molecular remodeling in the tumor microenvironment (TME) of melanoma and identify potential intervention targets to enhance the immunotherapeutic efficacy.

The TME is the biological environment that surrounds a tumor or cancer stem cell, composed of immune cells, blood vessels, extracellular matrix, fibroblasts, and lymphocytes, which are critical for tumor growth, treatment, and prognosis [9]. Huang et al. first identified a new subpopulation of immune cells, named TdLN‐TTSM cells, which belong to a branch of tumor‐specific CD8^+^ T cells and are crucially responsible for the response to immunotherapy [10]. However, the role of different immune cells or effector molecules in the efficacy of tumor‐targeted monoclonal antibody (mAb) therapy remains controversial [11, 12, 13]. Natural killer (NK) cell immunotherapy has rapidly developed in recent years and has become a popular research topic. For one of the focuses of debate, NK cell immunotherapy is the result of a global wave of tumor immunotherapy in the past 20 years [14, 15]. NK cells are important components of the natural immunity of the human body that play an important role in the occurrence and development of tumors. Similar to T cells, NK cells can be modified to better recognize specific tumors, can detect more tumor chemical signals than T cells, and are less likely to attack healthy tissue [16]. This indicates that treating patients with homologous NK cells reduces the risk of foreign cell attacks. The target cells of NK cells mainly include tumor cells, virus‐infected cells, autologous tissue cells, and parasites, which can effectively kill tumor cells and play an important role in inhibiting the occurrence, development, and spread of tumors; this is the basis of tumor cell and antibody immunotherapy [17, 18]. Although NK cells play an essential role in target cell killing and cytokine secretion, their heterogeneity remains poorly characterized.

Previous tissue genome and transcriptome data have greatly advanced the exploration of the biological processes of the melanoma immunotherapy response; however, the average aggregation of millions of cell signals deprives data on rare and unique cell subpopulations in the TME, which may be key to determining tumor initiation and progression [19, 20]. Single‐cell sequencing (scRNA‐seq) technology can be used to analyze the transcriptome data of each cell and better understand the key mechanisms of tumorigenesis and development [21]. The single‐cell atlas provides valuable insights into treatment response and drug resistance, and the identification of critical and rare cell subpopulations and molecular processes will help enhance the diagnosis of patients with cancer and accelerate the development of new drugs [22, 23, 24]. scRNA‐seq paves the way for the discovery of multidimensional biomarkers associated with immunotherapy response and resistance and will contribute to the development of next‐generation immunotherapies that may improve survival outcomes for patients with cancer [25, 26, 27]. Recently, spatial transcriptomics (ST) sequencing has yielded both in situ gene expression and spatial distribution data. By decoding the cellular and molecular basis of tumor diseases at the spatial resolution level, it is possible to analyze cell–cell interactions and their mechanisms in situ in tissues, which can not only provide information on tumor heterogeneity but also reveal the spatial composition of different cell populations [28]. This may be particularly relevant for immune checkpoint blocker (ICB) treatment. Therefore, there is a need to further explore the intrinsic characteristics of CM from other aspects, such as tumor heterogeneity and the immune microenvironment, through more accurate approaches, such as scRNA‐seq and ST, which will aid in explaining the differences in the response of patients with cancer immunotherapy. Immune cell types are subdivided into finer subclasses, forming an extremely complex TME that constantly interacts with itself and the tumor to co‐regulate it. However, the role of many cell subpopulations involved in tumor invasion and the underlying mechanisms of their interaction remain unexplored.

This study analyzed CM using scRNA‐seq to understand the various functions of tumor‐infiltrating immune cells, especially NK cells; elaborate on the clinical relevance of NK nuclear immunotherapy; and identify biomarkers of immunotherapeutic efficacy. Next, ST was used to understand the correlation between NK and melanoma cells and intercellular communications to provide new drug targets for melanoma treatment. Finally, based on the analysis of immune‐related genes in NK cells, two distinct immune subtypes were identified, each exhibiting unique immune microenvironment characteristics, prognostic implications, and potential therapeutic drugs.

## 2 Materials and Methods

### 2.1 Data Sources

The other melanoma scRNA‐seq datasets GSE115978, GSE72056, and GSE242477 and five melanoma on‐chip sequencing datasets (GSE7553, GSE15605, GSE19234, GSE19234, and GSE114445 [29, 30, 31]) were obtained from Gene Expression Omnibus (GEO). ST data from the dataset GSE229532 [32] were accessed via the UCSC Xena website, sourced from The Cancer Genome Atlas (TCGA). The TCGA dataset included bulk RNA‐seq data processed with log2(count+1), somatic cell mutation data processed with MuTect2, and clinical information for 103 patients with CM. Additionally, this study systematically gathered transcriptome datasets relevant to CM immunotherapy from studies by Hugo [19], Liu [33], Gide [34], Riaz [35], and Van Allen [36]. Ethical approval was obtained through the Ethics Committee of Nanfang hospital, Southern Medical University.

### 2.2 Single Cell Sequencing Raw Data Processing and Quality Control

The main steps are summarized below. Raw sequencing data were processed with a Cell Ranger for demultiplexing, alignment, and UMI matrix generation. Low‐quality cells (genes < 500 or > 6000; mitochondrial UMIs > 20%) were filtered using Seurat. Data underwent log‐normalization, selection of top 2000 variable genes, and batch correction via integration anchors. Principal components (PCs) were computed from integrated data, with the top 20 PCs selected by an elbow plot for downstream analysis. Cell clustering was performed (resolution = 0.8) and visualized in UMAP space, followed by annotation using canonical markers. Differential gene expression across clusters was identified via pairwise marker detection. Doublets were removed using Scrublet with standard thresholds.

### 2.3 Gene Set Variation Analysis (GSVA)

GSVA is a non‐parametric, unsupervised analysis method that evaluates gene set enrichment results in on‐chip transcriptomes. This method transforms the gene expression matrix among samples into a gene set expression matrix to determine whether various pathways are enriched in different samples. The gene set “h.all.v7.5.2.symbols” was obtained from the MSigDB database [37] and incorporated into single‐cell data for analysis. The characteristic genes of NK and melanoma cells were assessed using GSVA with default settings. Subsequently, the scores of the characteristic genes for myeloid, T, and B cells were computed across multiple datasets. A significance level of *p* < 0.05 was employed as the criterion for replicating the gene sets, with the GSVA conducted using the R package GSVA.

### 2.4 Evaluation of the Clinical Efficacy of Immunotherapy

The primary clinical endpoints examined in this study were the objective response rate (ORR) and overall survival (OS). ORR was evaluated across all cohorts using the Response Evaluation Criteria in Solid Tumors (RECIST, v1.1) [38]. Patients were stratified into two categories based on their response to treatment: those demonstrating complete (CR) or partial (PR) response were classified as responders (R), while those exhibiting stable (SD) or progressive (PD) disease were categorized as non‐responders (NR).

### 2.5 Assessment of Biological Functional Characteristics

To examine the biological functions among distinct cell subpopulations, the FindAllMarkers function in the R language was employed to discern differentially expressed genes across various cell types. Genes exhibiting a logFC ≥ 1 and a false discovery rate (FDR) < 0.05 were deemed specific upregulated genes for each respective cell subtypes. Gene Ontology (GO) analysis conducted comprehensive functional enrichment investigations on a large scale, encompassing biological processes, molecular functions, and cellular components. In this study, the clusterProfiler package in R was employed to conduct GO annotation analysis of the differentially expressed genes among patients exhibiting distinct unfolded protein response phenotypes, with statistical significance determined by an FDR threshold of < 0.05.

### 2.6 CRISPR Analysis

To investigate potential therapeutic targets for genes unique to NK cell subsets, data were collected from seven published CRISPR/Cas9 screening studies that evaluated the individual effects of gene knockout on tumor immunity. These studies include Freeman (2019), Kearney (2018), Manguso (2017), Pan (2018), Patel (2017), Vredevoogd (2019), and Lawson (2020). These studies were categorized into 17 datasets according to the model cell lines used and the therapeutic conditions employed. CRISPR analysis focused on melanoma, breast, colon, and kidney cancer cell lines. These data were used to identify genes across various datasets that may play a role in regulating lymphocyte‐mediated cancer cell destruction and affect the response to immunotherapy.

### 2.7 Subtype Analysis of Patients With CM

Based on the necroptosis characteristic genes and TCGA‐SKCM expression data, the k‐means method in the “ConsensusClusterPlus” R package [39] was used to perform unsupervised cluster analysis to identify the immune subtypes. The concordant clustering algorithm was used to determine the cluster numbers. The analysis was repeated 1000 times to ensure the stability of the categories. Survival analysis was performed after grouping to determine the influence of various subtypes on prognosis.

### 2.8 Somatic Mutation Analysis

Somatic mutation data from TCGA‐SKCM skin melanoma samples were acquired in “MAF” format. Somatic mutations were analyzed using the HMF somatic mutation workflow from https://github.com/hartwigmedical/pipeline [40]. Subsequently, the total number of somatic non‐synonymous point mutations within each sample and tumor mutational burden (TMB) were determined using maftools (v2.10.0) in the R software [37]. A waterfall plot was constructed using maftools to visually represent and synthesize the mutational landscape of all identified genes.

### 2.9 Deconvolution Analysis

SPOTlight analysis was performed for deconvolution analysis, as reported previously [41]. ST processes were deconvoluted using SPOTlight, integrating the scRNA‐seq cell‐type profile to ascertain spatial interactions. Default SPOTlight parameters were used in the analysis.

### 2.10 Analysis of Cell–Cell Communication

The CellChat (v1.1.3) R package [42] was frequently employed to analyze intercellular communication networks derived from single‐cell transcriptome sequencing data. CellChat was used to quantitatively deduce and examine intercellular communication networks from single‐cell RNA sequencing data, with a circle diagram illustrating cell interactions among melanoma cell subsets and a bubble diagram enumerating significant ligand pairs involved in intercellular signal transmission.

## 3 Results

### 3.1 Single‐Cell Transcriptome Atlas Revealed the Cellular Composition of CM

**FIGURE 1:**
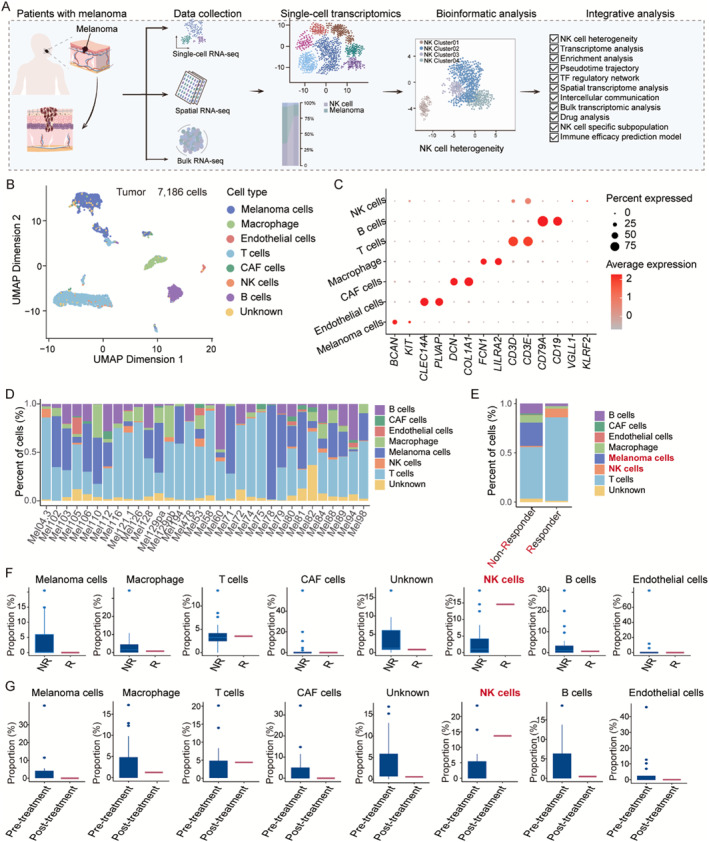
Single‐cell Atlas of human melanoma tissues. (A) Schematic representation of the study design. (B) UMAP plot demonstrating the cell distribution from cutaneous melanoma, color‐coded by the annotated cell types. (C) Expression matrix of cell‐type marker genes in seven cell types isolated from melanoma tissues. The dot size represents the percentage of cells expressing the genes in each cluster. The expression intensity of marker is shown. (D) Bar plots indicating the proportion of major cell types in each patient. (E) The plot showing cellular fraction of major cell types in response group and non‐response group. (F) Boxplot showing cellular fractions of major cell types in response group and non‐response group. (G) Boxplot showing cellular fractions of major cell types in pre‐treatment group and post‐treatment groups.

Immune checkpoint inhibitors (ICI) produce durable responses in some melanoma patients, but many patients derive no clinical benefit, and the molecular underpinnings of such resistance remain elusive. Here, we leveraged a single‐cell RNA‐seq dataset (GSE115978) from 32 melanoma tumors to interrogate malignant cell states that promote immune evasion. The flow of analysis is depicted in Figure 1A. Following quality control measures, a total of 7186 cells were identified. Using the R package Seurat, cluster analysis was conducted to categorize the cells into eight major cell types, namely, melanoma cells (*n* = 2018), B cells (*n* = 818), CAF (*n* = 106), endothelial cells (*n* = 104), macrophages (*n* = 420), NK cells (*n* = 92), T cells (*n* = 3321), and unknown cell types (*n* = 307), based on marker gene annotation (Figure 1B). Each cell type exhibited a high degree of specificity in the expression of their established marker genes (Figure 1C). Subsequent analysis was conducted to determine the distribution of the major cell types within each melanoma tissue, which indicated that the eight major cell types were present in nearly all patients, albeit within varying proportions (Figure 1D). The most abundant were melanoma cells, with the highest transcripts, consistent with their highly malignant characteristics, and T cells were the most abundant immune cell type. However, the proportions of NK and tumor cells always repelled each other in the same patient with melanoma. This phenomenon suggests that a significant correlation exists between NK cell infiltration and the clinical immune response to melanoma. The proportions of T and NK cells in the immunotherapy responder group were significantly higher, whereas the proportions of melanoma and B cells were relatively higher in the NR group, reflecting that immune function was suppressed in the NR group (Figure 1E). Figure 1F shows differences in cell types between the responder and NR groups; the results also show that in the responder group, the number of NK cells increased while that of melanoma cells decreased. Figure 1G displays differences in cell types between paired pre‐ and post‐treatment tumor tissues, revealing that the number of NK cells increased and that of melanoma cells decreased in the post‐treatment group. Although these differences in the above analysis results were not statistically significant, this statistical difference was most likely caused by an insufficient sample size. In summary, this study found that the expansion of NK cells in melanoma is associated with a better response to ICB; that is, NK cells may play a certain promoting role in sensitizing the immunotherapeutic efficacy of CM.

### 3.2 Heterogeneity and Characteristics of NK Cells in the Microenvironment of CM

**FIGURE 2:**
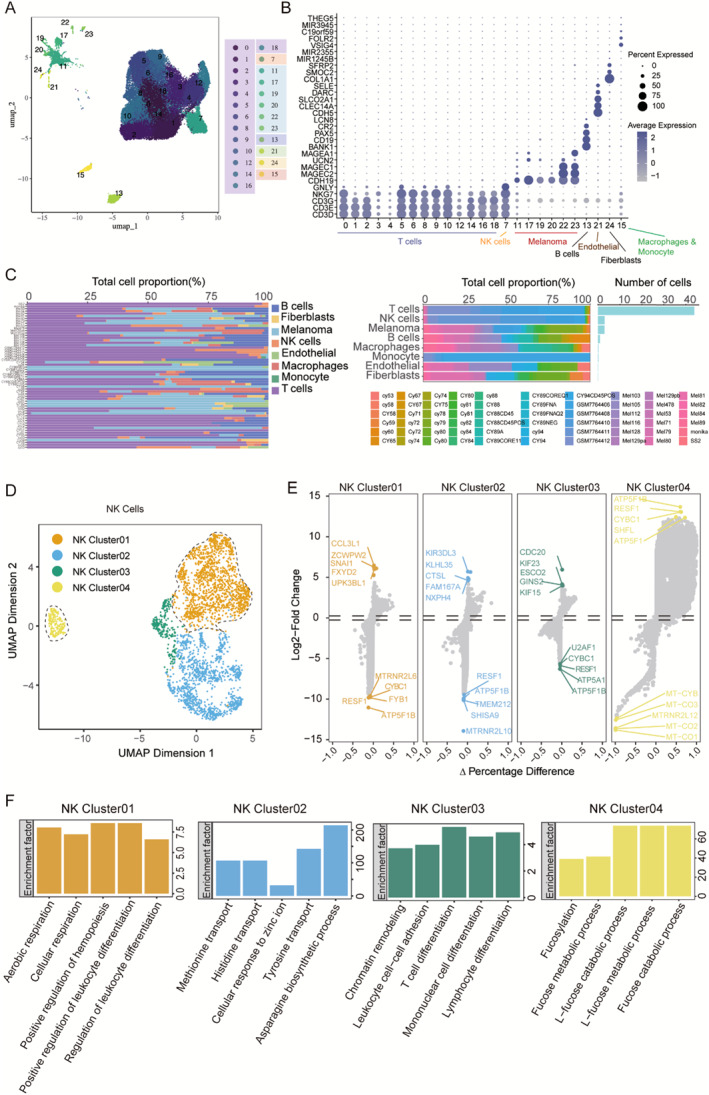
Re‐clustering and focused analysis of the NK cells. (A) UMAP plots of 51,616 cells from melanoma tissues, showing 25 clusters. (B) Dot plots showing average expression of cell‐type specific signature genes in indicated cell clusters. (C) Proportion of 8 major cell types showing in bar plots in different donors, tissues, and total cell number of each cell type are shown. (D) In the current investigation, UMAP projections of 3036 NK cells from melanoma patients revealed four significant NK cell subgroups. (E) Expression of characteristic marker genes in different NK cell subtypes. (F) The GO enrichment pathways in NK cell subsets are displayed in the bar chart.

To further verify the correlation between NK cells and immunotherapy efficacy, we also collected three melanoma single‐cell datasets, namely, GSE115978, GSE72056, and GSE242477, and combined them to remove the batch effect using the Harmony package. After quality control, a total of 51,616 cells were obtained. Cluster analysis of these cells was performed using the R package Seurat, and a total of 25 cell populations were obtained (Figure 2A). These cell populations were annotated by cell signature genes and divided into seven cell types, namely, T cells, NK cells, melanoma cells, B cells, endothelial cells, fibroblasts, and macrophages/monocytes (Figure 2B). Almost all cell populations were present in each individual lesion, and there were differences in the distribution of these cell types among patients, indicating the extreme heterogeneity of CM (Figure 2C).

Next, we identified the cell populations of the NK cell cluster using UMAP analysis. Figure 2D shows that NK cells were heterogeneous and clustered into four subgroups: NK cluster 01 (*n* = 1525), NK cluster 02 (*n* = 1073), NK cluster 03 (*n* = 249), and NK cluster 04 (*n* = 189). The four distinct NK subclusters shown in Figure 2E are consistent with the theory that NK cells have different identities and roles in the extracellular matrix. NK cluster 01 cells exhibited a high expression of the proinflammatory cytokine CCL3L1. NK cluster 02 was characterized by the presence of *KIR3DL3*, a regulator of cytotoxicity in human NK cells and certain T cells, with significant immunomodulatory capabilities. NK cluster 03 displayed elevated levels of *CDC20*, a critical player in the cell division cycle. NK cluster 04 showed increased expression of the canonical NK cell marker gene *ATP5F1B*. Notable differences were observed in the NK cell subsets among melanoma molecular subtypes and individuals with varying immune responses. GO analysis was performed to identify the biological functions of each NK cell type (Figure 2F). NK cluster 01 cells showed significantly enhanced mitochondrial function, including aerobic respiration, oxidative phosphorylation signals, and leukocyte differentiation‐related signals, which play an important role in the immune response, activation, and effect stages. NK cluster 02 cells played an important role in the enrichment of signals related to amino acid transport and asparagine biosynthesis, which play key roles in regulating T‐cell activation. NK cluster 03 cells were involved in signal enrichment related to T‐cells, monocytes, lymphocyte differentiation, and leukocyte adhesion, which play key roles in specific cellular immune response processes. NK cluster 04 cells were enriched in signaling related to the fucose metabolic process, which plays an important role in triggering antitumor immune cell activity mediated by CD4^+^ T cells.

### 3.3 NK Cells Correlation Pseudo‐Time Series Analysis Revealed Its Maturation Trajectory in Melanoma Tissues

**FIGURE 3:**
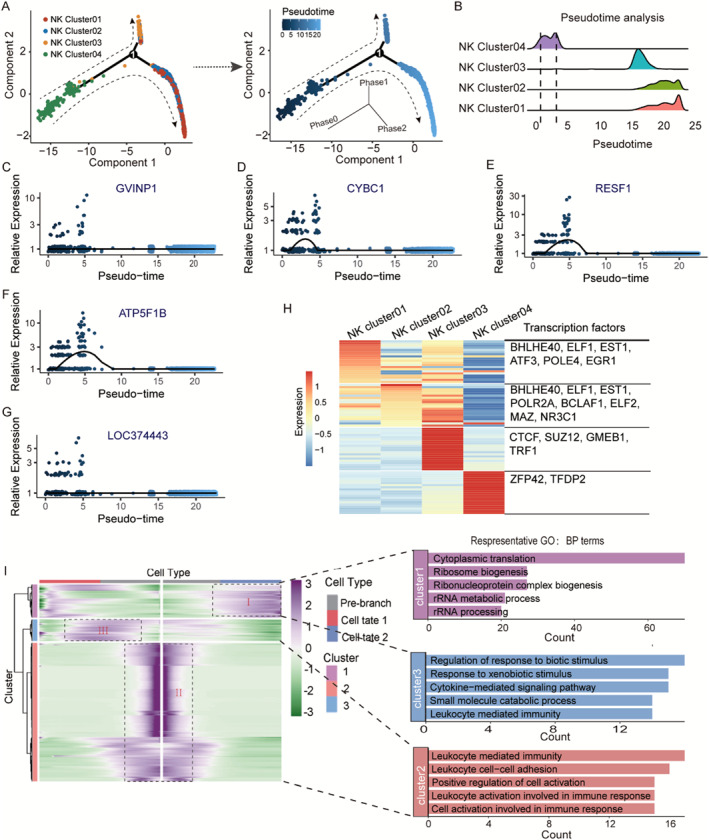
Differentiation trajectories of NK cells in melanoma tissues. (A) Differentiation trajectory of NK cells (3036 cells). (B) Pseudotime trajectory of NK cells. (C–G) The expression dynamics of representative genes along the pseudotime. (H) Heatmap shows relative expression of highly expressed transcription factors in each NK cell subtypes. (I) (Left) Heatmap shows the gene expression dynamics during NK cells differentiation. (Right) GO enrichment of the gene cluster with pseudo‐time variation. Genes (row) are clustered and cells (column) are ordered according to the pseudo‐time development.

All NK cell trajectories were analyzed using the R package Monocle2 to determine how they differentiated in the TME. NK cells can be divided into three categories at the pseudosequence level (Figure 3A). NK cluster 04 is in the very early stage of NK cell development, NK cluster 02 is in the transitional stage, and NK cluster 01 and NK cluster 03 are mainly in the final stage of NK cell development. Figure 3B also shows the developmental trajectory of NK cells. NK cluster 04 cells differentiate into two main branches: NK cluster 01 and NK cluster 03 cells. The expression of three genes—*CYBC1*, *RESF1*, and *ATP5F1B*—decreased from an initial high expression to the end and then gradually decreased (Figure 3C–G).

Single‐cell regulatory network inference and clustering (SCENIC) was applied to assess upstream transcription factors (regulons) driving the heterogeneity of NK cells [43]. Our findings suggest that Bhlhe40 plays a role in modulating the transcription factors of NK cluster 01 and NK cluster 02 cells, whereas CTCF may serve as the transcription factor governing NK cluster 03 cells (Figure 3H). We then conducted scRNA‐seq and pathway enrichment analysis along the track and found significant changes in the expression of 1825 genes. Figure 3I shows the differential heatmaps of gene expression at the three stages of NK cell evolution. In the initial stage of NK cell evolution, it was mainly enriched in “leukocyte‐mediated immunity” and “leukocyte cell–cell adhesion” related pathways. In the middle stage of NK cell evolution, it was mainly enriched in the “regulation of response to biotic stimulus” and “response to xenobiotic stimulus” pathways. The terminal stage of NK cell evolution was mainly enriched in the “cytoplasmic translation” and “ribosome biogenesis” and other related pathways, which were involved in NK cell protein synthesis.

### 3.4 ST Analysis Revealed Cell–Cell Communication in the Microenvironment of CM

**FIGURE 4:**
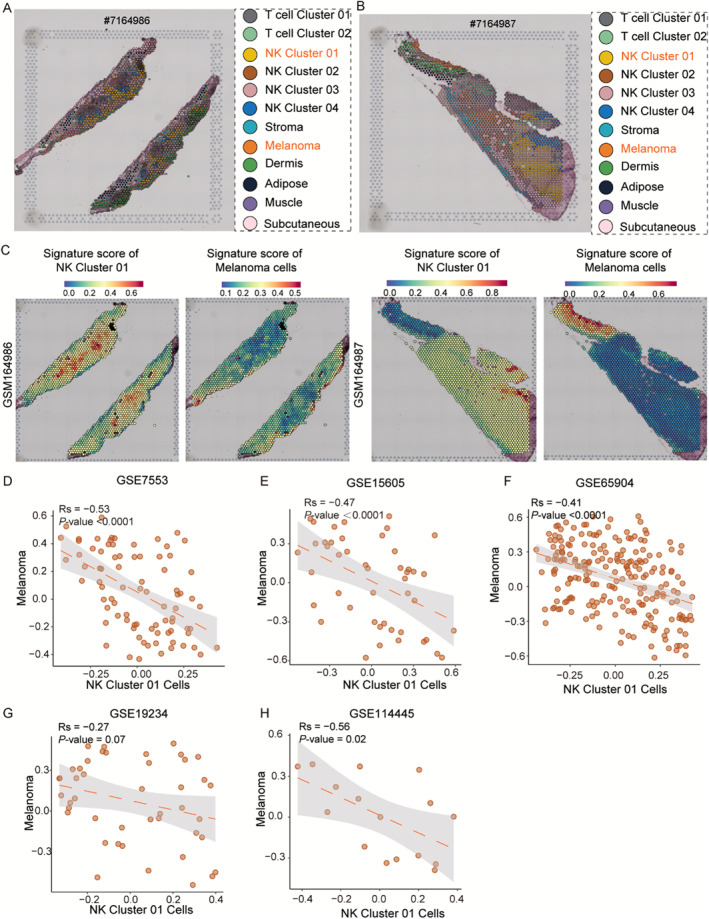
The positional relationship between NK cells and melanoma cells revealed by ST. (A–B) Unbiased clustering of ST spots and define cell types of each cluster. (C) Spatial feature plots of signature score of NK cluster 01 cells and melanoma cells in tissue sections. (D–H) The Pearson correlation of signature score of NK cluster 01 cells (*x* axis) and melanoma cells (*y* axis) in the GEO datasets.

To further assess the spatial organization of NK and melanoma cells, two ST samples were analyzed in CM. Based on unbiased clustering and spot features, we classified the spots into 12 clusters. The results of sample#7164986 showed that NK cluster 01 and melanoma cells had a certain co‐localization relationship, with a mutually exclusive relationship (Figure 4A). Similarly, sample # 7164987 also showed a certain co‐localization relationship between NK cluster 01 and melanoma cells, with fewer melanoma cells in the area with more NK cluster 01 cells, suggesting that NK cluster 01 cells may inhibit the presence of CM malignant cells (Figure 4B). In addition, the signature score of NK cluster 01 or melanoma cells showed a significant negative correlation (Figure 4C). We also observed variations in gene expression among cells located in distinct spatial regions (Supporting Information S1: Figure S1). These findings suggest that there is a crosstalk between the two cells in space.

Finally, we validated our results using other bulk melanoma datasets (GSE7553, *n* = 87; GSE15605, *n* = 74; GSE19234, *n* = 44; GSE65904, *n* = 214; and GSE114445, *n* = 34). The results indicated that NK cluster 01 cells were correlated with melanoma cells in the GSE7553 (*r* = −0.53, *p* < 0.001; Figure 4D), GSE15605 (*r* = −0.47, *p* < 0.001; Figure 4E), and GSE65904 (*r* = −0.41, *p* < 0.001; Figure 4F) datasets. In the GSE19234 dataset, NK cluster 01 cells showed a trend of correlation with melanoma cells (*r* = −0.27, *p* = 0.07; Figure 4G), and in the GSE114445 dataset also, NK cluster 01 cells were correlated with melanoma cells (*r* = −0.56, *p* = 0.02; Figure 4H).

### 3.5 Analysis of Cell Interaction Between NK and Melanoma Cells

**FIGURE 5:**
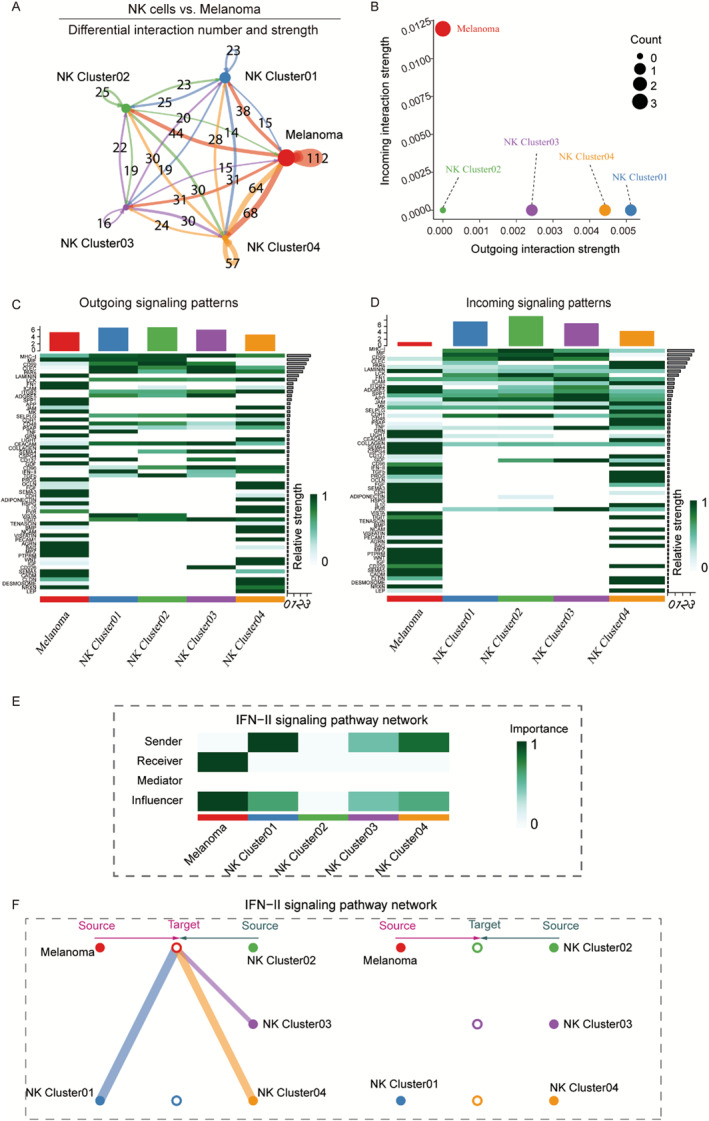
Inference of cell‐cell communications by CellChat shows global alterations in signaling pathways‐mediated communications between NK cells and melanoma cells. (A) Circle plots of the interaction quantity and interaction strength between NK cells and melanoma cells. Blue lines indicate that the displayed communication is decreased, whereas red lines indicate that the displayed communication is increased in melanoma. (B) Identification of signaling roles for cells using network centrality analysis. (C–D) Heatmaps of the overall (comprising both outgoing and incoming) signaling flows of each cell population medicated by individual signaling axes. (E) Heatmap of the IFN‐II signaling network displaying relative importance of each cell group ranked according to the computed four network centrality measures. (F) Hierarchical plot showing the interactions of NK cells and melanoma cells via IFN‐II signaling.

To identify the key mediators of interaction between tumor and NK cells, we inferred and quantified the communication relationship between different cells in melanoma samples using CellChat and focused on analyzing the receptor–ligand interaction relationship between NK cluster 01 and melanoma cells. CellChat analysis results showed that melanoma cells could function as both signal transmitters and receivers, mainly reflected in the fact that melanoma cells can activate various NK cluster cells and play a role in TME remodeling. Meanwhile, NK cluster 01 cells function as signal transmitters that, in turn, inhibit melanoma cells (Figure 5A,B). The overall incoming and outgoing signaling patterns are presented in Figure 5C,D. We showed that CD99, MHC‐I, and MIF signaling were activated in almost all cell types, indicating their extensive roles. The LAMININ, FN1, ADGRE5, SPP1, MK, CDH1, and APP signals mainly originated from melanoma cells and were received by multiple cell types, suggesting that melanoma cells play extensive roles in the ecosystem. The IFN‐II signals predominantly emanated from NK cluster 01 and were perceived by melanoma cells, suggesting their contribution to the activation of NK cell cytotoxicity and augmentation of antigen presentation. As shown in Figure 5E, the NK cluster 01 cell subgroup showed a strong ability to “output” signals, whereas CM malignant cells showed a strong ability to “receive” signals in the type II interferon (IFNII) signaling pathway. The IFNII family consists of a single gene product, IFNγ, produced primarily by T and NK cells, and acts on cell types that express the IFNγ receptor (IFNγR). This conserved IFNγ transcriptome response enhances the antitumor immune response.

In addition, melanoma and NK cells were successively set as potential target cells, and NK cluster 01, NK cluster 03, and NK cluster 04 cells could target melanoma cells through IFNII signaling, among which NK cluster 01 had the strongest signal effect. However, when NK cluster 02, NK cluster 03, and NK cluster 04 cells were set as potential target cells, no positive results were obtained (Figure 5F).

**FIGURE 6:**
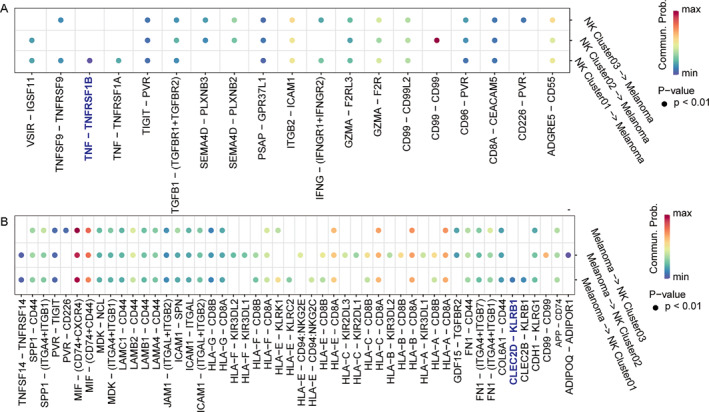
Bubble diagram of receptor ligand pair in interaction between NK cells and melanoma cells. (A) Receptor‐ligand pairs through which NK cells mediate regulatory effects on malignant cutaneous melanoma cells. (B) Receptor‐ligand pairs through which malignant cutaneous melanoma cells mediate regulatory effects on NK cells. Red and blue represent high and low probabilities of interaction, respectively.

To clarify the receptor–ligand regulatory relationship between NK cluster 01 and CM malignant cells, this study demonstrated the pairing of “input” and “output” signals, mainly from CM malignant cells, with the receptor–ligand of NK cluster 01 cells through CellChat analysis. Figure 6A shows that NK cluster 01 subtype cells “output” signals to regulate the receptor–ligand relationship in CM malignant cells. Thus, NK cluster 01 subtype cells have the greatest potential for regulating CM malignant cells through the tumor necrosis factor (TNF)–TNFRSF1B axis, suggesting that NK cluster 01 cells may respond to melanoma cells by producing proinflammatory cytokines, including IFNγ and TNF. Figure 6B shows the receptor–ligand relationship, indicating that NK cluster 01 subgroup cells receive signal regulation from CM malignant cells, indicating that CM malignant cells are most likely to receive and regulate NK cluster 01 cells through the CLEC2D–KLRB1 axis.

### 3.6 Gene Screening for NK Cluster 01‐Related Immunotherapeutic Efficacy

**FIGURE 7:**
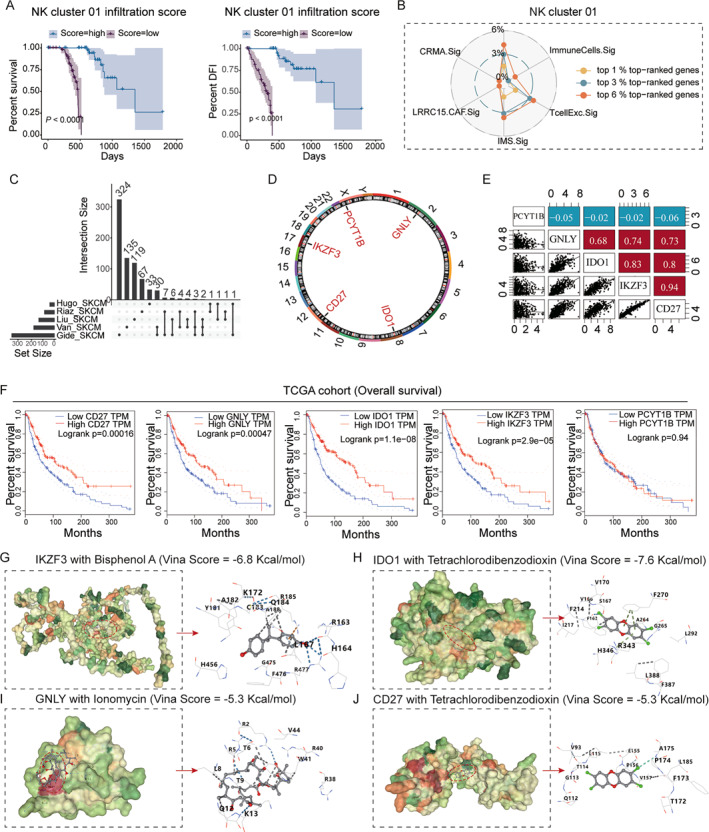
Genes linked to NK cluster 01 cell immune function and impact on prognosis. (A) Prognostic impact of tumor‐infiltrating NK cluster 01 cells on patients with melanoma. (B) A radar chart depicting the distribution of top‐ranking immune efficacy‐related genes within the NK cluster 01 cell subpopulation. (C) Upset map for the screening of immune efficacy‐associated genes across five GEO datasets. (D) Location of immune‐related genes on chromosomes. (E) Correlation analysis of immune‐related genes. (F) Effect of immunoefficacy related genes on the prognosis of melanoma patients. (G–J) 3D diagrams and interaction diagrams of molecular docking results. The blue bond is the hydrogen bond, the light blue bond is the weak hydrogen bond, the gray bond is the hydrophobic interaction, and the light green bond is the halogen bond. The yellow bond is ionic interaction, the orange bond is cation‐Pi interaction, and the dark green bond is Pi‐Pi interaction.

We found that NK cluster 01 cells were closely related to the immunotherapeutic efficacy in melanoma. To further verify this finding, a Kaplan–Meier plot was constructed. The results revealed that the higher the infiltration of NK cluster 01 cells in melanoma, the better the prognosis (Figure 7A). Next, we extracted specific genes from NK cluster 01 cells and compared them with previously published melanoma immunotherapy‐related tag genes in the CRISPR‐Cas9 dataset. We collected post‐knockout immune response data from seven CRISPR‐Cas9 cohorts and calculated the percentage of genes that ranked high in NK cluster 01 cells and previously published immune resistance markers, including TcellExc.Sig, ImmuneCells.Sig, IMS.Sig, LRRC15.CAF.Sig, and CRMA.Sig. The specific genes of NK cluster 01 cells had the highest percentage of the top genes, indicating that the specific genes of NK cluster 01 cells were most closely related to the immunotherapeutic efficacy (Figure 7B). To identify the genes most closely associated with the immunotherapeutic efficacy, the lasso‐logistic algorithm was used to screen the specific genes of the NK cluster 01 cell subtype in the five datasets, and the results were visualized using Upset maps (Figure 7C). Five candidate genes were identified: *GNLY*, *IDO1*, *CD27*, *IKZF3*, and *PCYT1B*. The chromosomal locations of these five genes are shown in Figure 7D. Correlation analysis indicated a statistically significant relationship between the identified genes (Figure 7E). We further analyzed the effect of the expression of these genes in CM tissues on patient prognosis, suggesting that these genes could be potential targets for the treatment of melanoma (Supporting Information S1: Figure S2, Figure 7F).

Small‐molecule compounds can interact with target biomacromolecules and inhibit or activate the biological functions of proteins. To identify potential activators, we predicted interactions between genes associated with immunological efficacy and drugs in the Comparative Toxicogenomics Database (CTD) database [44]. We used the AutoDock vina program on the CB‐Dock2 website to achieve blind docking and visualization of immunological efficacy–related genes with the corresponding small‐molecule compounds. CB‐Dock2 was used to perform molecular docking of four immunological efficacy–related genes with their corresponding active components, namely, IKZF3‐bisphenol A (Vina score = −6.8 Kcal/mol; Figure 7G), IDO1‐tetrachlorodibenzodioxin (Vina score = −7.6 Kcal/mol; Figure 7H), GNLY‐ionomycin (Vina score = −5.3 Kcal/mol; Figure 7I), CD27‐tetrachlorodibenzodioxin (Vina score = −5.3 Kcal/mol; Figure 7J). However, translational research of these small‐molecule drugs requires further investigation.

### 3.7 Construction and Validation of the Model for Predicting the Immunotherapeutic Efficacy in Melanoma

**FIGURE 8:**
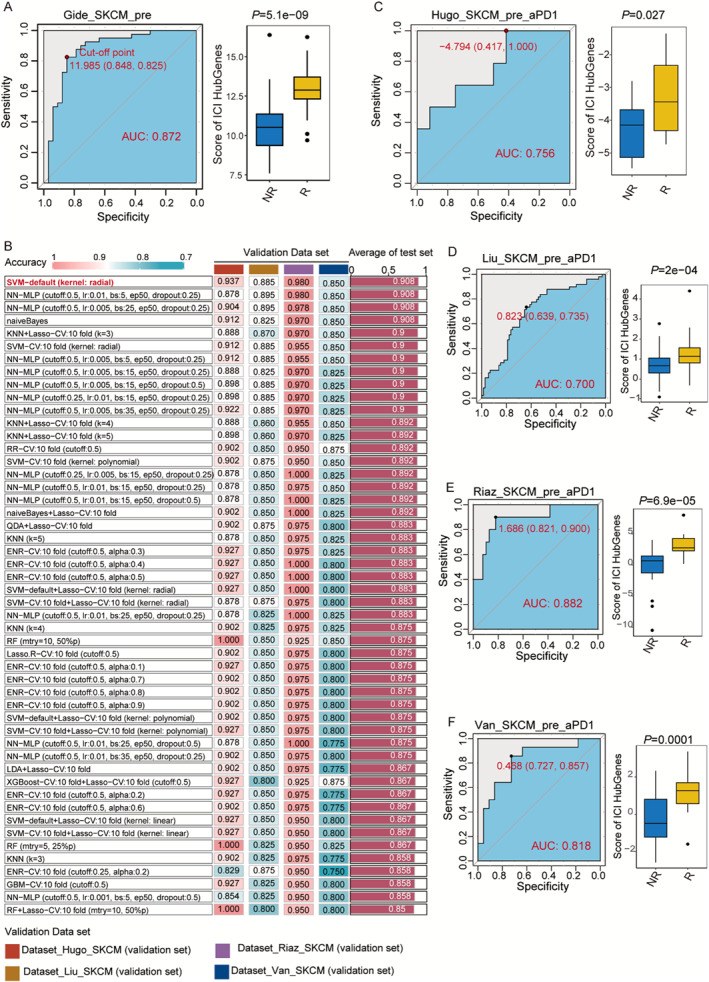
Construction and validation of immune efficacy prediction model for melanoma. (A) The plot shows the predictive performance of the scores of five melanoma immunoefficacy‐related genes in the Gide dataset, as well as the variation among different immunotherapeutic groups. (B) Fifty machine learning models were employed to forecast the immune response effectiveness in individuals with melanoma. (C–F) Validation of the immune efficacy prediction model in other datasets.

To identify the prediction ability of immunological efficacy of the five immunological efficacy–related genes, we used the Gide dataset as the training set and the remaining four datasets (Hugo, Liu, Riaz, and Van) as the validation set to verify the immunological efficacy prediction model. Figure 8A shows the construction scores of the five genes and their ability to predict immunological efficacy in the Gide dataset. The results showed that the area under the curve (AUC) score of the five genes was 0.872, indicating good predictive performance. In the responder group, the scores for the five genes were significantly higher (*p* = 5.1e‐09). Subsequently, to verify the prediction models of the five genes, 50 different machine learning algorithms were used to construct the models. The results showed that the receiver operating characteristic (ROC) curve of all 50 prediction model construction algorithms was > 0.85. The SVM‐default (kernel: radial) algorithm had the highest ROC, with an average of 0.908 across the four validation sets (Figure 8B). Additionally, the validation datasets Hugo, Liu, Riaz, and Van revealed findings that were consistent with those of the training set (Figure 8C–F). In short, the model constructed using these five genes had a high predictive value for the prediction of immunotherapeutic efficacy in melanoma.

### 3.8 Different Biological Functions, Survival, and Immune Microenvironment Patterns in Different Immune Subtypes

**FIGURE 9:**
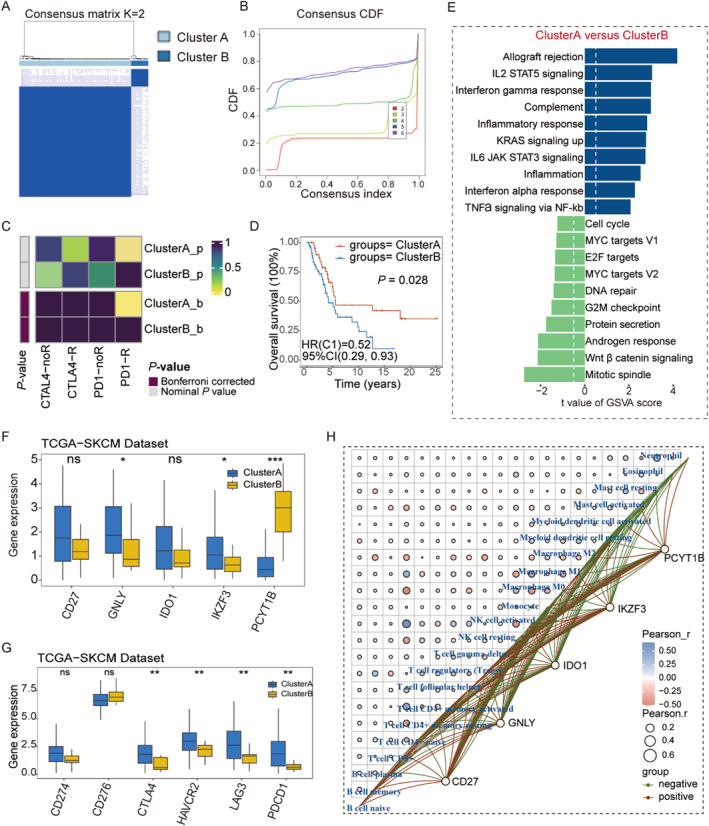
Unsupervised clustering analysis related to melanoma. (A) The consensus score matrix of all samples when *k* = 2. A higher consensus score between two samples indicates they are more likely to be grouped into the same cluster in different iterations. (B) The cumulative distribution functions of consensus matrix for each *k* (indicated by colors). (C) Submap analysis revealed that the different subtype was more sensitive to PD‐1 inhibitor. (D) Kaplan–Meier analysis for OS of the two subtypes in the melanoma cohort. (E) Differences in hallmark related pathway activities between cluster A and cluster B patients scored by the GSVA method. (F–G) The expressions of immune efficacy genes and immune checkpoint molecules were identified between cluster A and cluster B patients in the TCGA‐SKCM dataset. (H) Correlation between the abundance of immune cells and the expression of CD27, GNLY, IDO1, KZF3, and PCYT1B.

In this study, we performed an unsupervised clustering of five immunological efficacy–related genes for CM samples from the TCGA‐SKCM dataset (*n* = 103) and identified two clusters (Figure 9A). Figure 9B shows the consistency indices for different categories. The value of k was set to 2–6, and the results showed that *k* = 2 was the best parameter for dividing the 103 CM samples into subtypes A and B based on the five immunological efficacy–related genes. Submap analysis reported some differences in the immunotherapeutic efficacy between the two groups of patients, with patients in cluster A being more likely to benefit from immunotherapy than those in cluster B (Figure 9C). Kaplan–Meier survival analysis showed that patients in cluster A had a more favorable prognosis than those in cluster B (Figure 9D). Hallmark‐related pathway enrichment analysis showed that the main biological function enrichment in cluster A was mainly in immunoinflammatory signaling pathways, including interleukin (IL)‐2/STAT5 signaling, inflammatory response, and IL‐6/JAK/STAT3 signaling. In contrast, the major biological functions in cluster B were concentrated in proliferative signaling pathways, including the mitotic spindle, Wnt/β‐catenin signaling, and cell cycle (Figure 9E). Figure 9F shows that *GNLY* and *IKZF3* expression was significantly increased in cluster A (*p* < 0.05), whereas *PCYT1B* was highly expressed in cluster B (*p* < 0.0001). Although the difference in *CD27* and *IDO1* expression between the two groups was not statistically significant, they showed a trend of higher expression in cluster A. Additionally, six immunological checkpoint–related genes were explored in the two subtypes; *CTLA4*, *HAVCR2*, *LAG3*, and *PDCD1* were highly expressed in cluster A, which, to some extent, proved that cluster A was more likely to benefit from immunotherapy (Figure 9G). Finally, we identified the immune‐activated and immune‐resistant subtypes. In contrast to patients in cluster B, those in cluster A exhibited a higher prevalence of M1 macrophages and a lower prevalence of M2 macrophages (Figure 9H). This finding aligns with prior research indicating that INF‐γ has the capacity to promote M1‐type polarization in macrophages. Furthermore, patients classified in cluster A exhibited a higher proportion of activated NK cells and a lower proportion of resting NK cells. These findings indicate distinct immune infiltration profiles between the two subtypes. Specifically, patients in cluster A demonstrated a predisposition toward immune inflammation, whereas those in cluster B displayed a tendency toward immune desert.

### 3.9 Somatic Mutation Landscape and Druggable Categories Analysis of Different Melanoma Subgroups

**FIGURE 10:**
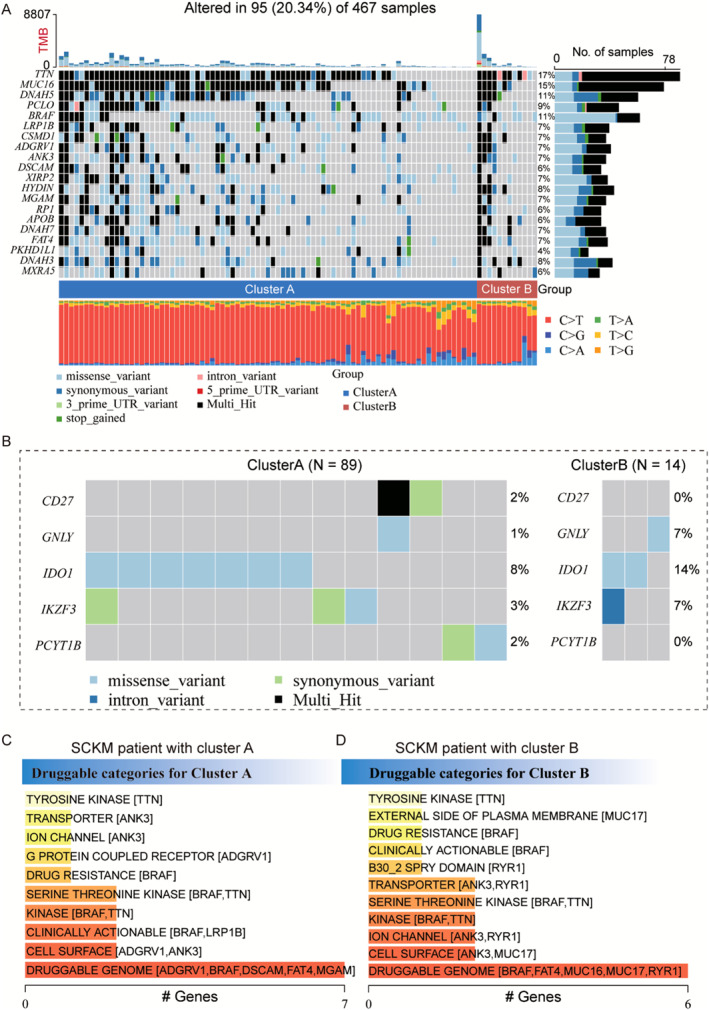
Genome‐wide mutation profiling in patients with melanoma and drug‐gene interactions. (A) Mutation landscape of cluster A and cluster B subtypes. The 20 genes with the highest mutation frequency are shown and samples are sorted by the TMB in each subtype. The small figure above shows the TMB, the numbers on the right exhibit the mutation frequency of each regulator, and the figure laterally shows the proportion of each variant. (B) Distribution of mutation frequency of CD27, GNLY, IDO1, IKZF3, and PCYT1B genes among cluster A and cluster B subgroups. (C–D) Drug–gene interactions, potential druggable gene categories based on the mutation genes in cluster A and cluster B samples.

To better identify the correlation between the characteristics of different melanoma populations and clinical treatment, the characteristics of somatic mutations in the subgroup population were evaluated, which revealed that somatic mutations were more frequent in patients in cluster A (Figure 10A). Mutations in *TTN*, *MUC16*, and *DNAH5* were higher in patients in cluster A than in those in cluster B. Patients in cluster A had a higher tumor mutation load and were likely to produce more neoantigens, resulting in patients benefiting more from immunotherapy, which also verified that inflammatory tumors were more sensitive to immune checkpoint inhibitors. Figure 10B shows the mutations in the five key genes of the melanoma subgroups.

Pharmacogenomics involves exploring the influence of genetic variations in genes on the therapeutic effect of drugs from the perspective of the genome. We examined the influence of genetic mutations on drug susceptibility. Figure 10C,D show the potential druggable gene categories along with the top five genes involved. Multiple drug classes were found to correlate with the mutation status of five key gene mutations in the TCGA‐SKCM study patients, particularly in the druggable genome.

**FIGURE 11:**
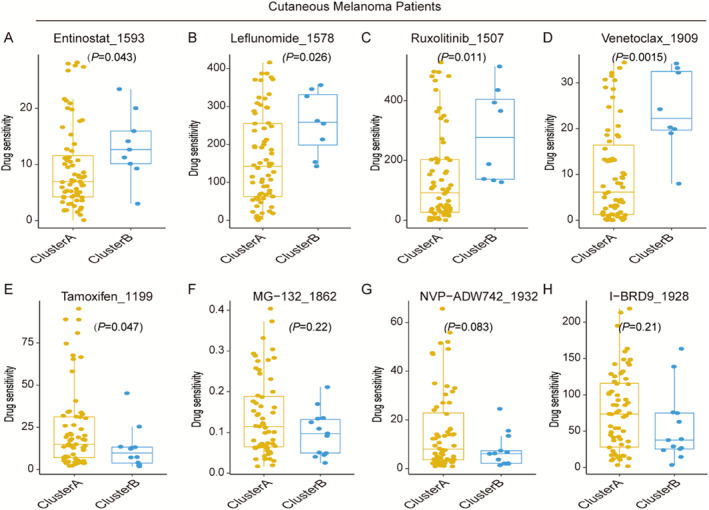
The predicted IC_50_ for drugs in the cluster A and B group. (A–H) The cluster A group was related to a lower IC_50_ in Entinostat_1593, Leflunomide_1578, Ruxolitinib_1507 and Venetoclax_1909, while the cluster B group was related to a lower IC_50_ in Tamoxifen_1199, MG‐132_1862, NVP‐ADW742_1932 and I‐BRD9_1928 (*p* < 0.05 by Wilcoxon test).

The Genomics of Drug Sensitivity in Cancer (GDSC) database was used to analyze the relationship between drug sensitivity and different melanoma subtypes. The results revealed that patients in cluster A were sensitive to entinostat_1593 (*p* = 0.043), leflunomide_1578 (*p* = 0.026), ruxolitinib_1507 (*p* = 0.011), and venetoclax_1909 (*p* = 0.0015; Figure 11A–D). Conversely, patients in cluster B were only sensitive to tamoxifen 1199 (*p* = 0.047) but were resistant to most drugs, including MG‐132_1862 (*p* = 0.22), NVPADW742_1932 (*p* = 0.083), and NVPADW742_1932 (*p* = 0.21; Figure 11E–H).

## 4 Discussions

Despite the recent remarkable success of cancer immunotherapy [45], its effectiveness varies among patients, and the detailed mechanism remains unclear. Additionally, in clinical trials of patients with melanoma receiving ICB therapy, relatively limited information has been obtained on predictive biomarkers and immunotherapy strategies. Therefore, it is important to identify the heterogeneity of the TME of melanoma, predict the early efficacy of ICB therapies, and screen individuals who are likely to benefit from ICB therapies. Through integrated analyses of scRNA‐seq, bulk RNA‐seq, and ST, our study provides a comprehensive picture of the landscape of the TME at single‐cell resolution and demonstrates that the interaction between NK cluster 01 and melanoma cells may play an instructive role in the responsiveness of ICB therapies. We found that the interaction between NK cluster 01 and melanoma cells is critical in the mechanism of immunotherapy response and proposed that NK cells could be a potential therapeutic target. We identified a panel of five genes as predicted biomarkers for screening the dominant populations that could benefit from immunotherapy. This compensates for the deficiency of several contemporary predictors, including PD‐L1 expression, tumor mutation burden, and microsatellite instability, which fail to sufficiently address this issue.

Tumor immune cell therapy is promising because of its high specificity and minimal side effects [46]. In addition to CAR T‐cell therapy, NK cell therapy, a new type of cancer cell therapy, has received considerable attention. NK cells can decide whether to kill tumor cells based on the balance of signals between active and inhibitory receptors on the cell surface [47]. When NK cells are activated, they release cytokines (such as IFNγ and TNF) and chemokines (such as macrophage inflammatory protein‐1α [MIP‐1α]), which, in turn, act on other immune cells such as macrophages and dendritic cells [48]. Although NK cells play an essential role in target cell killing and cytokine secretion, their heterogeneity remains poorly characterized. Tang et al. systematically identified five subgroups of NK cells at the pan‐cancer level and detailed the phenotypic and functional diversity of each group [49]. NK cell subpopulations in different developmental states are simultaneously present in the tumor, suggesting that NK cell migration to the tumor may be decoupled from NK cell maturation [49]. In this study, we systematically deciphered the heterogeneity and features of NK cells and demonstrated the dramatic remodeling of stromal compartments upon ICB therapy. We identified four subtypes of NK cells with different biological functions. We identified a key NK cell subpopulation, NK cluster 01, that influences the immune response to melanoma. One of the most significant features of NK cluster 01 cells was the expression of *CCL3L1*, which plays a crucial role in immune regulation and a defense role by producing MIP‐1α. We found that the increase in NK cluster 01 cells may contribute to the formation of an immune‐promoting microenvironment in melanoma, mainly manifested by an increase in M1‐type macrophages, reshaping the tumor immune microenvironment. Further evidence is required to validate the role of NK cluster 01 cells in melanoma. Therefore, targeting NK cells is a potential treatment option for melanomas.

NK cells can be divided into three categories at the pseudosequence level. NK cluster 04 is in the early stage of NK cell development, NK cluster 02 is in the transitional stage, and NK cluster 01 and NK cluster 03 are mainly in the final stage of NK cell development. The process of NK cell development involves a series of distinct stages, each characterized by the presence of specific surface markers. The prevailing model in current research posits that NK cells originate from CD34^+^CD45RA^+^ hematopoietic progenitor cells (HPCs) in the bone marrow [50]. In the bone marrow, NK cells develop from HPCs via common lymphoid progenitor cells (CLPs) and NK cell precursors and then migrate to the peripheral blood (conventional NK cells) or tissue (tissue‐resident NK cells). Recent evidence challenges this view and raises the possibility of more branched development, in which both CLPs and common myeloid progenitors can produce NK cell precursors [51]. Moreover, different precursor populations independently develop into different subpopulations of mature NK cells. NK cells are highly heterogeneous even within the same organs and tissues [52]. Because NK cells are a heterogeneous group, they are composed of subgroups with different phenotypes and functions in different tissue microenvironments. Therefore, a comprehensive and systematic study of NK cells in tumor tissues is necessary to clarify the obstacles to NK cell immunotherapy. The migration of NK cell subpopulations in tumors is regulated by chemokines produced in the TME and the expression profile of NK cell chemokine receptors. NK cell maturation is mediated by a variety of cytokines, of which IL‐15 plays a key role in the development of ordinary lymphoid progenitors into NK cells [53, 54].

Little is known about the dynamics of NK cell–mediated melanoma clearance. Vyas et al. reported that NK cells control lung metastasis of melanoma cells through different mechanisms, in which NK cells play a key role in shaping T‐cell–mediated in situ control of lung seed cancer cells [55]. Jiao et al. found that radiofrequency radiation reshapes the tumor immune microenvironment into an antitumor phenotype in pulmonary metastatic melanoma by inducing the active transformation of tumor‐infiltrating NK cells [56]. ST technology is rapidly developing and has been widely used to construct spatial organization maps and characterize the spatial and temporal heterogeneity of tissues. In this study, we found that NK cluster 01 and melanoma cells communicate with each other through an IFNII signaling pathway network and are mutually exclusive. The IFNγ signaling pathway is a double‐edged sword in immune monitoring [57]. On the one hand, CD8^+^T cells can inhibit the proliferation of tumor cells and enhance immune activity through IFNγ secretion. On the other hand, IFNγ derived from T cells can upregulate PD‐L1 expression on the surface of tumor cells to protect tumor cells from attack by immune cells.

Immunotherapeutic efficacy in the clinical setting is closely associated with the use of biomarkers. Biomarkers such as PD‐L1 expression, TMB, mismatch repair, and microsatellite instability have been identified as predictors of immunotherapy response to some extent [58, 59, 60, 61]. However, the current understanding of immunotherapy biomarkers remains incomplete, and existing biomarkers are not yet able to accurately identify the optimal patient population. IFNγ expression is commonly recognized as a predictive indicator of the effectiveness of anti–programmed cell death protein 1 (PD‐1)/PD‐L1 therapy [62]. Recently, increasing attention has been paid to the predictive value of intestinal microbiota. Analyzing the composition of the intestinal microbiota will help predict the therapeutic effects of anti‐PD‐1/PD‐L1 mAb [63]. Coana et al. demonstrated that NK cells have potential as prognostic biomarkers for predicting the response to PD‐1 inhibitors in patients with advanced melanoma [64]. In fact, no single factor is sensitive and specific enough to be used to screen patients who may benefit from PD‐1/PD‐L1–targeted therapy. Therefore, in our study, based on data analysis, we screened five genes derived from NK cells as a panel to predict immunotherapeutic efficacy, which revealed that the clinical response to immunotherapy could be accurately predicted using these genes. Multiple indicators were summarized for prediction and verified using multiple datasets to obtain valuable prediction indicators. However, further studies with larger cohorts are required to confirm these findings.

In short, this study provides a better understanding of the tumor ecosystem heterogeneity between immunotherapy responders and NR in terms of immune and tumor phenotypes. We emphasized the diversity of NK cells in melanoma, demonstrated the significant role of NK cluster 01 cells in enhancing tumor immunity at both the single‐cell and ST levels, and investigated the signaling interactions between NK cluster 01 and melanoma cells. Finally, we successfully established a biomarker utilizing the composition of the five genes for the purpose of screening dominant populations that can benefit from immunotherapy. The findings of this research are expected to offer valuable insights into the appropriateness and efficacy of immunotherapy in melanoma treatment.

## Author Contributions

**Zhicheng Hu:** data curation, validation. **Yongfei Chen:** formal analysis. **Hao Yang:** formal analysis, project administration. **Qiuming Pan:** investigation. **Hongrui Li:** data curation, investigation. **Shuting Li:** investigation. **Junxi Wang:** project administration, validation. **Yudi Huang:** resources, supervision. **Guanglong Huang:** conceptualization, writing – review and editing. **Shanqiang Qu:** conceptualization, funding acquisition, visualization, writing – original draft.

## Ethics Statement

Ethical approval was obtained through the Ethics Committee of Nanfang Hospital, Southern Medical University.

## Consent

Informed consent was exempted.

## Conflicts of Interest

The authors declare no conflicts of interest.

## Supporting information


Supporting Information S1

## Data Availability

All data and materials generated and analyzed during the present study are available from the corresponding author (qushq3@163.com) upon reasonable request.

## References

[1] E. Erdei and S. M. Torres , “A New Understanding in the Epidemiology of Melanoma,” Expert Review of Anticancer Therapy 10 (2010): 1811–1823. 21080806 10.1586/era.10.170 PMC3074354

[2] D. Ramírez‐Gamboa , A. L. Díaz‐Zamorano , E. R. Meléndez‐Sánchez , et al., “Photolyase Production and Current Applications: A Review,” Molecules 27 (2022): 5998. 36144740 10.3390/molecules27185998 PMC9505440

[3] K. A. Cronin , S. Scott , A. U. Firth , et al., “Annual Report to the Nation on the Status of Cancer, Part 1: National Cancer Statistics,” Cancer 128 (2022): 4251–4284. 36301149 10.1002/cncr.34479 PMC10092838

[4] W. W. Dzwierzynski , “Melanoma Risk Factors and Prevention,” Clinics in Plastic Surgery 48 (2021): 543–550. 34503715 10.1016/j.cps.2021.05.001

[5] S. Caksa , U. Baqai , and A. E. Aplin , “The Future of Targeted Kinase Inhibitors in Melanoma,” Pharmacology & Therapeutics 239 (2022): 108200. 35513054 10.1016/j.pharmthera.2022.108200 PMC10187889

[6] C. Jandus , D. Speiser , and P. Romero , “Recent Advances and Hurdles in Melanoma Immunotherapy,” Pigment Cell & Melanoma Research 22 (2009): 711–723. 19735459 10.1111/j.1755-148X.2009.00634.x

[7] J. Larkin , V. Chiarion‐Sileni , R. Gonzalez , et al., “Five‐Year Survival With Combined Nivolumab and Ipilimumab in Advanced Melanoma,” New England Journal of Medicine 381 (2019): 1535–1546. 31562797 10.1056/NEJMoa1910836

[8] Y. Ding , C. Liu , Y. Wu , and F. Fu , “Overcoming Melanoma Resistance to Immune Checkpoint Blockade Therapy Using Nano‐Strategies,” Biomedical Technology 4 (2023): 41–49.

[9] Y. Huang , H. Fan , and H. Ti , “Tumor Microenvironment Reprogramming by Nanomedicine to Enhance the Effect of Tumor Immunotherapy,” Asian Journal of Pharmaceutical Sciences 19 (2024): 100902. 38595331 10.1016/j.ajps.2024.100902 PMC11002556

[10] Q. Huang , X. Wu , Z. Wang , et al., “The Primordial Differentiation of Tumor‐Specific Memory CD8^+^ T Cells as Bona Fide Responders to PD‐1/PD‐L1 Blockade in Draining Lymph Nodes,” Cell 185 (2022): 4049–4066. 36208623 10.1016/j.cell.2022.09.020

[11] M. Wei , D. Shen , S. Mulmi Shrestha , J. Liu , J. Zhang , and Y. Yin , “The Progress of T Cell Immunity Related to Prognosis in Gastric Cancer,” BioMed Research International 2018 (2018): 3201940. 29682534 10.1155/2018/3201940 PMC5848132

[12] G. F. Ma , Q. Miao , Y. M. Liu , et al., “High FoxP3 Expression in Tumour Cells Predicts Better Survival in Gastric Cancer and Its Role in Tumour Microenvironment,” British Journal of Cancer 110 (2014): 1552–1560. 24548868 10.1038/bjc.2014.47 PMC3960619

[13] J. Hou , Z. Yu , R. Xiang , et al., “Correlation Between Infiltration of FOXP3^+^ Regulatory T Cells and Expression of B7‐H1 in the Tumor Tissues of Gastric Cancer,” Experimental and Molecular Pathology 96 (2014): 284–291. 24657498 10.1016/j.yexmp.2014.03.005

[14] T. D. Stenger and J. S. Miller , “Therapeutic Approaches to Enhance Natural Killer Cell Cytotoxicity,” Frontiers in Immunology 15 (2024): 1356666. 38545115 10.3389/fimmu.2024.1356666 PMC10966407

[15] C. Forbes , S. Nierkens , and A. M. Cornel , “Thymic NK‐Cells and Their Potential in Cancer Immunotherapy,” ImmunoTargets and Therapy 13 (2024): 183–194. 38558927 10.2147/ITT.S441639 PMC10979679

[16] M. Jakóbisiak , W. Lasek , and J. Gołab , “Natural Mechanisms Protecting Against Cancer,” Immunology Letters 90 (2003): 103–122. 14687712 10.1016/j.imlet.2003.08.005

[17] M. A. Caligiuri , “Human Natural Killer Cells,” Blood 112 (2008): 461–469. 18650461 10.1182/blood-2007-09-077438 PMC2481557

[18] G. Tabellini , O. Patrizi , K. Dobbs , et al., “From Natural Killer Cell Receptor Discovery to Characterization of Natural Killer Cell Defects in Primary Immunodeficiencies,” Frontiers in Immunology 10 (2019): 1757. 31396241 10.3389/fimmu.2019.01757 PMC6668486

[19] W. Hugo , J. M. Zaretsky , L. Sun , et al., “Genomic and Transcriptomic Features of Response to Anti‐PD‐1 Therapy in Metastatic Melanoma,” Cell 165 (2016): 35–44. 26997480 10.1016/j.cell.2016.02.065 PMC4808437

[20] W. Deng , Y. Ma , Z. Su , et al., “Single‐Cell RNA‐Sequencing Analyses Identify Heterogeneity of CD8^+^ T Cell Subpopulations and Novel Therapy Targets in Melanoma,” Molecular Therapy Oncolytics 20 (2021): 105–118. 33575475 10.1016/j.omto.2020.12.003 PMC7851490

[21] Q. Ye , H. Meng , F. Ye , et al., “Single‐Cell RNA Sequencing of Pediatric Renal Tissues Revealed the Potential Relationship Between Immunoglobulin A Nephropathy and Immunoglobulin A Vasculitis With Nephritis,” Clinical and Translational Medicine 14 (2024): e1651. 38591641 10.1002/ctm2.1651 PMC11002991

[22] S. Pellecchia , M. Franchini , G. Viscido , R. Arnese , and G. Gambardella , “Single Cell Lineage Tracing Reveals Clonal Dynamics of Anti‐EGFR Therapy Resistance in Triple Negative Breast Cancer,” Genome Medicine 16 (2024): 55. 38605363 10.1186/s13073-024-01327-2 PMC11008053

[23] Y. Yao , Z. Chen , Q. Wu , Y. Lu , X. Zhou , and X. Zhu , “Single‐Cell RNA Sequencing of Retina Revealed Novel Transcriptional Landscape in High Myopia and Underlying cell‐type‐specific Mechanisms,” MedComm 4 (2023): e372. 37746666 10.1002/mco2.372 PMC10511833

[24] S. Cheng , Y. Zou , M. Zhang , et al., “Single‐Cell RNA Sequencing Reveals the Heterogeneity and Intercellular Communication of Hepatic Stellate Cells and Macrophages During Liver Fibrosis,” MedComm 4 (2023): e378. 37724132 10.1002/mco2.378 PMC10505372

[25] Z. Wang , K. L. Kirkwood , Y. Wang , et al., “Analysis of the Effect of CCR7 on the Microenvironment of Mouse Oral Squamous Cell Carcinoma by Single‐Cell RNA Sequencing Technology,” Journal of Experimental & Clinical Cancer Research 43 (2024): 94. 38539232 10.1186/s13046-024-03013-y PMC10976828

[26] D. Jovic , X. Liang , H. Zeng , L. Lin , F. Xu , and Y. Luo , “Single‐Cell RNA Sequencing Technologies and Applications: A Brief Overview,” Clinical and Translational Medicine 12 (2022): e694. 35352511 10.1002/ctm2.694 PMC8964935

[27] H. F. Sun , L. D. Li , I. W. Lao , et al., “Single‐Cell RNA Sequencing Reveals Cellular and Molecular Reprograming Landscape of Gliomas and Lung Cancer Brain Metastases,” Clinical and Translational Medicine 12 (2022): e1101. 36336787 10.1002/ctm2.1101 PMC9637666

[28] Z. Zhong , J. Hou , Z. Yao , et al., “Domain Generalization Enables General Cancer Cell Annotation in Single‐Cell and Spatial Transcriptomics,” Nature Communications 15 (2024): 1929. 10.1038/s41467-024-46413-6 PMC10908802 38431724

[29] L. Raskin , D. R. Fullen , T. J. Giordano , et al., “Transcriptome Profiling Identifies HMGA2 as a Biomarker of Melanoma Progression and Prognosis,” Journal of Investigative Dermatology 133 (2013): 2585–2592. 23633021 10.1038/jid.2013.197 PMC4267221

[30] D. Bogunovic , D. W. O'Neill , I. Belitskaya‐Levy , et al., “Immune Profile and Mitotic Index of Metastatic Melanoma Lesions Enhance Clinical Staging in Predicting Patient Survival,” Proceedings of the National Academy of Sciences of the United States of America 106 (2009): 20429–20434. 19915147 10.1073/pnas.0905139106 PMC2787158

[31] B. Y. Yan , S. Garcet , N. Gulati , et al., “Novel Immune Signatures Associated With Dysplastic Naevi and Primary Cutaneous Melanoma in Human Skin,” Experimental Dermatology 28 (2019): 35–44. 30326165 10.1111/exd.13805 PMC6333525

[32] D. Filipescu , S. Carcamo , A. Agarwal , et al., “MacroH2A Restricts Inflammatory Gene Expression in Melanoma Cancer‐Associated Fibroblasts by Coordinating Chromatin Looping,” Nature Cell Biology 25 (2023): 1332–1345. 37605008 10.1038/s41556-023-01208-7 PMC10495263

[33] D. Liu , B. Schilling , D. Liu , et al., “Integrative Molecular and Clinical Modeling of Clinical Outcomes to PD1 Blockade in Patients With Metastatic Melanoma,” Nature Medicine 25 (2019): 1916–1927. 10.1038/s41591-019-0654-5 PMC6898788 31792460

[34] T. N. Gide , C. Quek , A. M. Menzies , et al., “Distinct Immune Cell Populations Define Response to Anti‐PD‐1 Monotherapy and Anti‐PD‐1/Anti‐CTLA‐4 Combined Therapy,” Cancer Cell 35 (2019): 238–255. 30753825 10.1016/j.ccell.2019.01.003

[35] N. Riaz , J. J. Havel , V. Makarov , et al., “Tumor and Microenvironment Evolution During Immunotherapy With Nivolumab,” Cell 171 (2017): 934–949. 29033130 10.1016/j.cell.2017.09.028 PMC5685550

[36] E. M. Van Allen , D. Miao , B. Schilling , et al., “Genomic Correlates of Response to CTLA‐4 Blockade in Metastatic Melanoma,” Science 350 (2015): 207–211. 26359337 10.1126/science.aad0095 PMC5054517

[37] A. Liberzon , A. Subramanian , R. Pinchback , H. Thorvaldsdóttir , P. Tamayo , and J. P. Mesirov , “Molecular Signatures Database (MSigDB) 3.0,” Bioinformatics 27 (2011): 1739–1740. 21546393 10.1093/bioinformatics/btr260 PMC3106198

[38] J. S. Lee , H. J. Choi , B. K. Kim , et al., “The Modified Response Evaluation Criteria in Solid Tumors (RECIST) Yield a More Accurate Prognoses than the RECIST 1.1 in Hepatocellular Carcinoma Treated With Transarterial Radioembolization,” Gut Liver 14 (2020): 765–774. 32050313 10.5009/gnl19197 PMC7667935

[39] M. D. Wilkerson and D. N. Hayes , “ConsensusClusterPlus: A Class Discovery Tool With Confidence Assessments and Item Tracking,” Bioinformatics 26 (2010): 1572–1573. 20427518 10.1093/bioinformatics/btq170 PMC2881355

[40] L. C. Demmers , K. Kretzschmar , A. Van Hoeck , et al., “Single‐Cell Derived Tumor Organoids Display Diversity in HLA Class I Peptide Presentation,” Nature Communications 11 (2020): 5338. 10.1038/s41467-020-19142-9 PMC7577990 33087703

[41] M. Elosua‐Bayes , P. Nieto , E. Mereu , I. Gut , and H. Heyn , “SPOTlight: Seeded NMF Regression to Deconvolute Spatial Transcriptomics Spots With Single‐Cell Transcriptomes,” Nucleic Acids Research 49 (2021): e50. 33544846 10.1093/nar/gkab043 PMC8136778

[42] S. Jin , C. F. Guerrero‐Juarez , L. Zhang , et al., “Inference and Analysis of Cell‐Cell Communication Using CellChat,” Nature Communications 12 (2021): 1088. 10.1038/s41467-021-21246-9 PMC7889871 33597522

[43] S. Aibar , C. B. González‐Blas , T. Moerman , et al., “SCENIC: Single‐cell Regulatory Network Inference and Clustering,” Nature Methods 14 (2017): 1083–1086. 28991892 10.1038/nmeth.4463 PMC5937676

[44] A. P. Davis , C. J. Grondin , R. J. Johnson , et al., “The Comparative Toxicogenomics Database: Update 2017,” Nucleic Acids Research 45 (2017): D972–D978. 27651457 10.1093/nar/gkw838 PMC5210612

[45] E. Michaels , N. Chen , and R. Nanda , “The Role of Immunotherapy in Triple‐Negative Breast Cancer (TNBC),” Clinical Breast Cancer 24 (2024): 263. 38582617 10.1016/j.clbc.2024.03.001

[46] D. Mutailifu , A. Aini , and A. Maimaitiaili , “Integrated Bioinformatics Analysis and Machine Learning Approach for the Identification of Immune‐Related Genes in the Diagnosis of Aortic Valve Calcification With Periodontitis,” Biomedical Technology 10 (2025): 100087.

[47] J. A. Yoder , T. M. Orcutt , D. Traver , and G. W. Litman , “Structural Characteristics of Zebrafish Orthologs of Adaptor Molecules That Associate With Transmembrane Immune Receptors,” Gene 401 (2007): 154–164. 17719728 10.1016/j.gene.2007.07.014 PMC2049010

[48] Y. T. Bryceson , M. E. March , H. G. Ljunggren , and E. O. Long , “Activation, Coactivation, and Costimulation of Resting Human Natural Killer Cells,” Immunological Reviews 214 (2006): 73–91. 17100877 10.1111/j.1600-065X.2006.00457.x PMC3845883

[49] F. Tang , J. Li , L. Qi , et al., “A Pan‐Cancer Single‐Cell Panorama of Human Natural Killer Cells,” Cell 186 (2023): 4235–4251. 37607536 10.1016/j.cell.2023.07.034

[50] J. W. Fathman , D. Bhattacharya , M. A. Inlay , J. Seita , H. Karsunky , and I. L. Weissman , “Identification of the Earliest Natural Killer Cell‐Committed Progenitor in Murine Bone Marrow,” Blood 118 (2011): 5439–5447. 21931117 10.1182/blood-2011-04-348912 PMC3217348

[51] D. Gotthardt , J. Trifinopoulos , V. Sexl , and E. M. Putz , “JAK/STAT Cytokine Signaling at the Crossroad of NK Cell Development and Maturation,” Frontiers in Immunology 10 (2019): 2590. 31781102 10.3389/fimmu.2019.02590 PMC6861185

[52] Y. Simoni , M. Fehlings , H. N. Kløverpris , et al., “Human Innate Lymphoid Cell Subsets Possess Tissue‐Type Based Heterogeneity in Phenotype and Frequency,” Immunity 46 (2017): 148–161. 27986455 10.1016/j.immuni.2016.11.005 PMC7612935

[53] D. G. Hesslein and L. L. Lanier , “Transcriptional Control of Natural Killer Cell Development and Function,” Advances in Immunology 109 (2011): 45–85. 21569912 10.1016/B978-0-12-387664-5.00002-9

[54] E. Mrózek , P. Anderson , and M. A. Caligiuri , “Role of Interleukin‐15 in the Development of Human CD56+ Natural Killer Cells From CD34+ Hematopoietic Progenitor Cells,” Blood 87 (1996): 2632–2640. 8639878

[55] M. Vyas , M. Requesens , T. H. Nguyen , D. Peigney , M. Azin , and S. Demehri , “Natural Killer Cells Suppress Cancer Metastasis by Eliminating Circulating Cancer Cells,” Frontiers in Immunology 13 (2022): 1098445. 36733396 10.3389/fimmu.2022.1098445 PMC9887278

[56] J. Z. Jiao , Y. Zhang , W. J. Zhang , et al., “Radiofrequency Radiation Reshapes Tumor Immune Microenvironment Into Antitumor Phenotype in Pulmonary Metastatic Melanoma by Inducing Active Transformation of Tumor‐Infiltrating CD8^+^ T and NK Cells,” Acta Pharmacologica Sinica 45 (2024): 1492. 38538718 10.1038/s41401-024-01260-5 PMC11192955

[57] M. R. Zaidi and G. Merlino , “The Two Faces of Interferon‐γ in Cancer,” Clinical Cancer Research 17 (2011): 6118–6124. 21705455 10.1158/1078-0432.CCR-11-0482 PMC3186825

[58] M. Reck , D. Rodríguez‐Abreu , A. G. Robinson , et al., “Pembrolizumab Versus Chemotherapy for PD‐L1‐Positive Non‐Small‐Cell Lung Cancer,” New England Journal of Medicine 375 (2016): 1823–1833. 27718847 10.1056/NEJMoa1606774

[59] M. D. Hellmann , T. E. Ciuleanu , A. Pluzanski , et al., “Nivolumab Plus Ipilimumab in Lung Cancer With a High Tumor Mutational Burden,” New England Journal of Medicine 378 (2018): 2093–2104. 29658845 10.1056/NEJMoa1801946 PMC7193684

[60] M. J. Overman , R. McDermott , J. L. Leach , et al., “Nivolumab in Patients With Metastatic DNA Mismatch Repair‐Deficient or Microsatellite Instability‐High Colorectal Cancer (CheckMate 142): An Open‐Label, Multicentre, Phase 2 Study,” Lancet Oncology 18 (2017): 1182–1191. 28734759 10.1016/S1470-2045(17)30422-9 PMC6207072

[61] D. P. Carbone , M. Reck , L. Paz‐Ares , et al., “First‐Line Nivolumab in Stage IV or Recurrent Non‐Small‐Cell Lung Cancer,” New England Journal of Medicine 376 (2017): 2415–2426. 28636851 10.1056/NEJMoa1613493 PMC6487310

[62] A. V. Balar , D. Castellano , P. H. O'Donnell , et al., “First‐Line Pembrolizumab in cisplatin‐Ineligible Patients With Locally Advanced and Unresectable or Metastatic Urothelial Cancer (KEYNOTE‐052): A Multicentre, Single‐Arm, Phase 2 Study,” Lancet Oncology 18 (2017): 1483–1492. 28967485 10.1016/S1470-2045(17)30616-2

[63] L. Xia , L. Oyang , J. Lin , et al., “The Cancer Metabolic Reprogramming and Immune Response,” Molecular Cancer 20 (2021): 28. 33546704 10.1186/s12943-021-01316-8 PMC7863491

[64] Y. Pico de Coaña , M. Wolodarski , I. van der Haar Àvila , et al., “PD‐1 Checkpoint Blockade in Advanced Melanoma Patients: NK Cells, Monocytic Subsets and Host PD‐L1 Expression as Predictive Biomarker Candidates,” OncoImmunology 9 (2020): 1786888. 32939320 10.1080/2162402X.2020.1786888 PMC7470181

