# Supplementary material for: Integrating Single‐Cell and Spatial Transcriptomics Reveals NK Cell Subpopulations Associated With Immunotherapy for Melanoma
Source: Smart Med. 2025 Dec 3;4(4):e70023. doi: 10.1002/smmd.70023 (PMC12677594; doi:10.1002/smmd.70023)
Supplement: Supplementary file 1 — Supporting Information S1 [file SMMD-4-e70023-s001.docx]

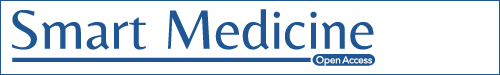


*Supplementary Materials*

**Materials and Methods**

**1 | Single cell sequencing raw data processing and quality control**

Cell Ranger (version 2.2.0) is utilized for the processing of raw data, multiplexing of cell barcodes, mapping of reads to the transcriptome, and downsampling of reads. Normalized aggregated data is generated between samples as required. These procedures result in the creation of a basic unique molecular identifier (UMI) count matrix, which is subsequently transformed into Seurat objects using the R package Seurat (version 4.0.6) ^1^. Cells exhibiting UMI counts less than 500 or mitochondria-derived UMI counts exceeding 20% are classified as low-quality cells and are excluded. Additionally, single cells with over 6,000 detected genes are filtered out to eliminate potential duplicates.

Following quality control procedures, the UMI count matrix undergoes logarithmic normalization. Subsequently, the top 2,000 variable genes are utilized to generate potential Anchors through the Seurat FindIntegration Anchors function. The IntegrateData function is then applied to merge the data, resulting in a new matrix containing 2,000 features with batch effects regressed out. To reduce the dimensionality of the single-cell RNA sequencing dataset, principal component analysis (PCA) is conducted on the integrated data matrix ^2^. The first 20 principal components (PCs) were extracted using Seurat's Elbowplot function and utilized for subsequent analysis. Major cell clusters were identified using Seurat's FindClusters feature with a resolution parameter set to the default value of 0.8, followed by visualization using UMAP. Known biological cell types were assigned to individual cells based on conventional markers from prior research studies. Differential gene expression analysis between cell clusters was conducted using Seurat's FindAllMarkers function. The comprehensive analysis and integration of single-cell data was conducted utilizing the R software Seurat. Quality control for dual-cell analysis was executed using the R language Scrublet package, whereby cells with fewer than 300 sequenced genes were removed, along with those exhibiting more than 20% mitochondrial gene reads. Normalization and standardization of individual sample data were achieved through principal component analysis, while batch effects between samples were addressed using the Harmony package. The Uniform Manifold Approximation and Projection (UMAP) algorithm was employed to reduce dimensions and visually represent single cell data.

**2 | Spatial transcriptome data analysis**

Spatial transcriptome data was processed and analyzed utilizing the Python package Scanpy (version 1.9.1) ^3^. Spatial coordinates exceeding 20,000, expressed genes surpassing 6,000, and mitochondrial genes constituting more than 10% were filtered. Normalization of count and spatial information data was conducted through the normalize total function, followed by extraction of the first 2000 highly variable genes using the highly_variable_genes function. Subsequently, principal component analysis (PCA), uniform manifold approximation and projection (UMAP), and the Leiden algorithm were employed.

**3 | Screening of predictors of melanoma immunotherapy efficacy and training and parameter adjustment of predictive models**

To investigate genes associated with immune response in melanoma, this study utilized 1000 Lasso regression models to screen five melanoma datasets for genes within the NK Cluster 01. The results were integrated using an Upset map, identifying genes common to two datasets as predictors of melanoma immunotherapy efficacy. The Gide 2019 dataset was employed as the training set, while the remaining four datasets served as validation sets for the development of melanoma immunotherapy prediction models.

In this study, a classification model for ICB response was trained using various Machine Learning methods, such as Support Vector Machine (SVM), Naive Bayes (NB), Random Forest (RF), K-Nearest Neighbors (KNN), AdaBoost classification trees (Ada-Boost), enhanced Logistic regression (LogiBoost), and Logistic regression models. Each ML algorithm was optimized using fivefold Cross-Validation (CV) for hyperparameter adjustment. To ensure robustness, the optimization process was repeated 10 times for each individual parameter.

**4 | Molecular docking**

To investigate the potential interaction mechanism between genes associated with melanoma immunoefficacy and small drug molecules, the CTD database ^4^ was utilized to predict gene-drug interactions, followed by the selection of small drug molecules exhibiting the highest affinity with genes related to melanoma immunoefficacy. Subsequently, the CB-Dock2 website ^5^ was employed for molecular docking studies involving genes linked to melanoma immunoefficacy and their respective small molecule compounds. The CB-Dock2 website represents an enhanced iteration of the CB-Dock server, specifically designed for the blind docking of protein ligands. This platform seamlessly integrates cavity detection, docking procedures, and homologous template fitting to accurately predict binding sites and affinity between proteins and ligands based on their respective three-dimensional structures. Such capabilities facilitate computer-aided drug discovery efforts by leveraging the structural information obtained from the PubChem database for the molecular structure of the drug ^6^. Following this, X-ray crystal structures of genes linked to melanoma immunoefficacy were acquired from the Protein Data Bank (PDB) protein structure database ^7^. In cases where genes lacked X-ray structures in the PDB Protein Structure Database, the projected structure was retrieved from AlphaFoldDB (AlphaFold Protein Structure Database) ^8^. The Predicted Local Distance Difference Test (pLDDT) per residue for the primary domains of the structure exceeded 70. Structures with a pLDDT value below 50 indicate low confidence in the prediction. In the range of 50 < pLDDT < 70, the prediction structure is characterized by moderate confidence, while in the range of 70 < pLDDT < 90, the prediction structure exhibits high confidence. Moreover, when pLDDT exceeds 90, the predicted structure demonstrates high confidence. Subsequently, the AutoDock Vina program ^9^ from the CB-Dock2 website was employed to conduct blind docking and visualize interactions between melanoma immunoefficacy related genes and corresponding small molecule compounds.

**5 | Trajectory and Pseudo-time Analysis**

To elucidate the developmental trajectories of NK cells, the R software package Monocle2 was employed to analyze the pseudo-time trajectories of individual cells. The functions newCellDataSet(), estimateSizeFactors(), and estimateDispersions() were employed for these analyses. The detectGenes() function was used to filter out cells expressing low quality, with the parameter “min_expr = 0.1” set accordingly.

**6 | Statistical analysis**

Data processing and analysis were conducted using R software (v4.1.3). The statistical significance of normally distributed variables in the comparison of two sets of continuous variables was assessed using the independent Student’s t-test, whereas the difference between non-normally distributed variables was analyzed using the Mann-Whitney U test (Wilcoxon rank-sum test). Chi-square or Fisher’s exact tests were used to compare and analyze the statistical significance between two sets of categorical variables. The R survival package was used for survival analysis, Kaplan–Meier survival curves to illustrate survival disparities, and log-rank test to evaluate the significance of the variance in survival time between the two cohorts. A *P*-value < 0.05 indicated the threshold for determining statistically significant outcomes.


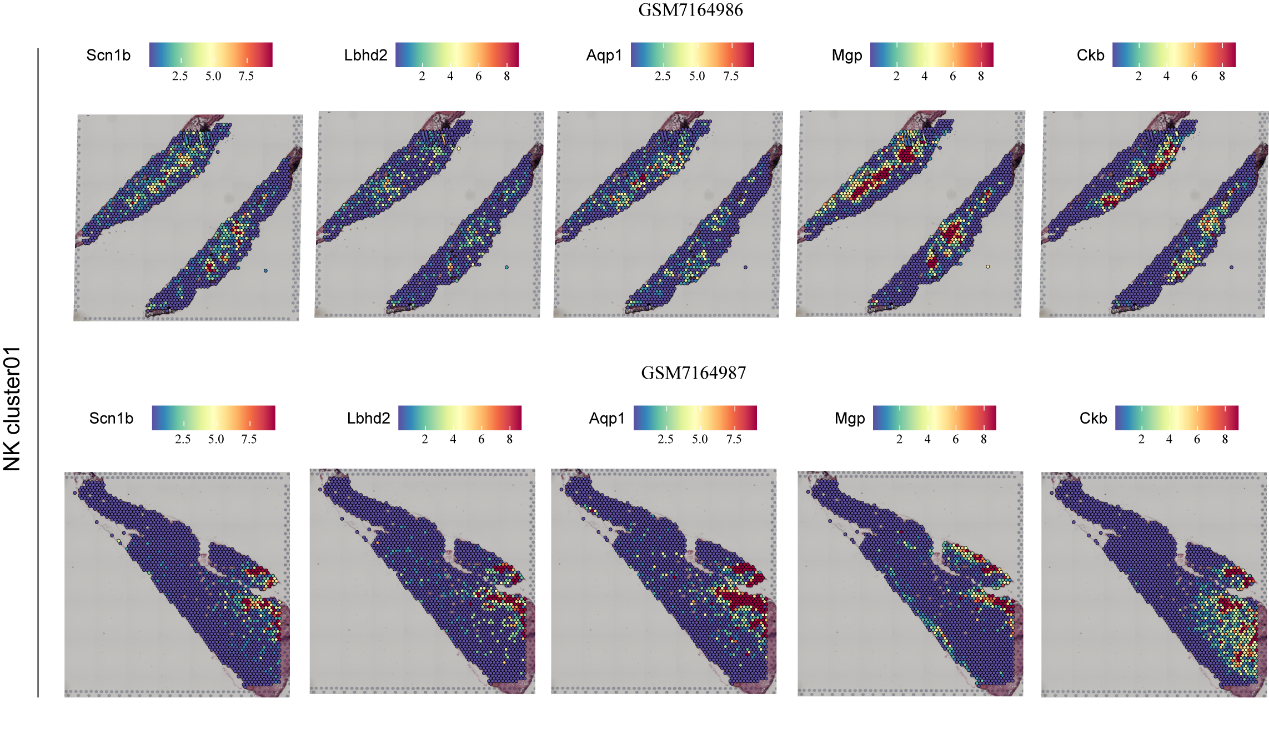


Figure S1. Spatial feature plots of gene expression of Scn1b, Lbhd2, Aqp1, Mgp, and Ckb in tissue sections of GSM7164986 and GSM7164987.


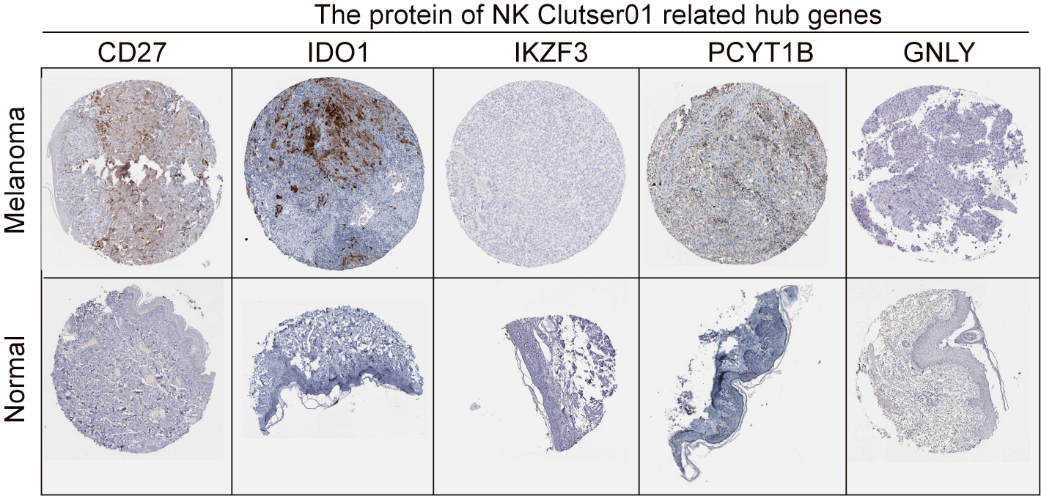


Figure S2. The Human Protein Atlas database analysis results show the expression levels of CD27, IDO1, IKZF3, PCYT1B, and GNLY proteins in melanoma and normal skin tissues.

**References**

1 Hao Y., Hao, S., Andersen-Nissen, E., et al. Integrated analysis of multimodal single-cell data. *Cell* 2021;184: 3573-3587.e3529.

2 Lever Jake, Krzywinski, Martin, and Altman, Naomi. Principal component analysis. *Nature methods* 2017;14: 641-642.

3 Wolf F. A., Angerer, P., and Theis, F. J. SCANPY: large-scale single-cell gene expression data analysis. *Genome biology* 2018;19: 15.

4 Davis A. P., Wiegers, T. C., Johnson, R. J., et al. Comparative Toxicogenomics Database (CTD): update 2023. *Nucleic acids research* 2023;51: D1257-d1262.

5 Liu Y., Yang, X., Gan, J., et al. CB-Dock2: improved protein-ligand blind docking by integrating cavity detection, docking and homologous template fitting. *Nucleic acids research* 2022;50: W159-w164.

6 Kim S., Chen, J., Cheng, T., et al. PubChem 2023 update. *Nucleic acids research* 2023;51: D1373-d1380.

7 Protein Data Bank: the single global archive for 3D macromolecular structure data. *Nucleic acids research* 2019;47: D520-d528.

8 Varadi M., Anyango, S., Deshpande, M., et al. AlphaFold Protein Structure Database: massively expanding the structural coverage of protein-sequence space with high-accuracy models. *Nucleic acids research* 2022;50: D439-d444.

9 Eberhardt J., Santos-Martins, D., Tillack, A. F., et al. AutoDock Vina 1.2.0: New Docking Methods, Expanded Force Field, and Python Bindings. *Journal of chemical information and modeling* 2021;61: 3891-3898.
